# Supplemental Light-Emitting Diode Inter-Lighting Increases Tomato Fruit Growth Through Enhanced Photosynthetic Light Use Efficiency and Modulated Root Activity

**DOI:** 10.3389/fpls.2019.01656

**Published:** 2020-01-10

**Authors:** Martina Paponov, Dmitry Kechasov, Jozef Lacek, Michel J. Verheul, Ivan A. Paponov

**Affiliations:** ^1^ Division of Food Production and Society, Norwegian Institute of Bioeconomy Research (NIBIO), Ås, Norway; ^2^ Institute of Experimental Botany, Czech Academy of Sciences, Prague, Czechia

**Keywords:** light-emitting diode, tomato, xylem sap, photosynthesis, fruit growth, diurnal rhythm

## Abstract

We investigated the effect of supplemental LED inter-lighting (80% red, 20% blue; 70 W m^−2^; light period 04:00–22:00) on the productivity and physiological traits of tomato plants (Flavance F1) grown in an industrial greenhouse with high pressure sodium (HPS) lamps (235 W m^−2^, 420 µmol m^−2^ s^−1^ at canopy). Physiological trait measurements included diurnal photosynthesis and fruit relative growth rates, fruit weight at specific positions in the truss, root pressure, xylem sap hormone and ion compositions, and fruit quality. In the control treatment with HPS lamps alone, the ratio of far-red to red light (FR:R) was 1.2 at the top of the canopy and increased to 5.4 at the bottom. The supplemental LED inter-lighting decreased the FR:R ratio at the middle and low positions in the canopy and was associated with greener leaves and higher photosynthetic light use efficiency (PLUE) in the leaves in the lower canopy. The use of LED inter-lighting increased the biomass and yield by increasing the fruit weight and enhancing plant growth. The PLUE of plants receiving supplemental LED light decreased at the end of the light period, indicating that photosynthesis of the supplemented plants at the end of the day might be limited by sink capacity. The supplemental LED lighting increased the size of fruits in the middle and distal positions of the truss, resulting in a more even size for each fruit in the truss. Diurnal analysis of fruit growth showed that fruits grew more quickly during the night on the plants receiving LED light than on unsupplemented control plants. This faster fruit growth during the night was related to an increased root pressure. The LED treatment also increased the xylem levels of the phytohormone jasmonate. Supplemental LED inter-lighting increased tomato fruit weight without affecting the total soluble solid contents in fruits by increasing the total assimilates available for fruit growth and by enhancing root activity through an increase in root pressure and water supply to support fruit growth during the night.

## Introduction

Light is the main factor that limits the year-round production of greenhouse-grown tomatoes in northern latitudes. For this reason, sustainable production of greenhouse tomatoes depends heavily on supplemental lighting. For the same reason, any increase in the effectiveness of supplementary lighting will also increase crop yields and the efficiency of energy use, thereby providing economic benefits to greenhouse tomato producers.

The current primary source of supplementary greenhouse lighting is top lighting using high-pressure sodium (HPS) lamps. However, the delivery of light from the top of the greenhouse creates a strong light gradient along the high-wiring canopy of crops like tomato, so irradiance at the bottom of the canopy is quite low (Gomez et al., 2013). The disadvantage of this light gradient relates to the curvilinear response of leaf photosynthesis to light intensity, which reduces the photosynthetic light use efficiency (PLUE, photosynthesis per unit captured photon) of the uppermost leaves (Terashima et al., 2005). By contrast, the low light intensity at the bottom of the canopy and accompanying leaf senescence strongly restricts photosynthesis in the lower leaves (Acock et al., 1978; Xu et al., 1997), which are an important source of assimilates for the growing fruits as well as for the roots (Wardlaw, 1990).

One method for diminishing the strong light gradient along the canopy is to use inter-lighting systems, where lamps are placed between the plant rows to provide adequate illumination along the entire length of the plant canopy (Davis and Burns, 2016; Bantis et al., 2018). Light-emitting diode (LED) lamps are the preferred lighting source, as they have a low operating temperature and do not produce significant amounts of radiant heat (Singh et al., 2015). This low heat production is important, since the lamps are located between the plant rows and in close proximity to the leaves.

Intra-canopy lighting stimulates photosynthetic rates in the lower-canopy leaves and prevents their premature senescence (Pettersen et al., 2010; Dueck et al., 2011; Trouwborst et al., 2011; Gomez and Mitchell, 2016). These responses to supplemental inter-lighting might be driven both by the increased light intensity in the lower canopy and by modulation of the light spectrum by the LED lamps. In a canopy without inter-lighting, the strong absorbance of red light by chlorophyll and the reflection and transmission of far-red light results in a significant increase in the far-red to red (FR:R) ratio from the top to the bottom of the canopy. For example, investigation with dense crop stands of cowpea (Frantz et al., 2000) showed an approximately 12-fold increase in the FR:R (730:600 nm) ratio from the upper to the lower canopy. Supplemental LED inter-lighting can decrease the FR:R ratio along the canopy because most of the LED lamps currently used for inter-lighting are constructed with red and blue diodes to match the red and blue wavelengths that are most efficiently absorbed by photosynthetic pigments (Chory, 2010). This inter-lighting can also increase the light intensity in the blue spectrum, with potential effects on plant responses driven by phytochromes, cryptochrome, and phototropin blue-light receptors (Lin, 2002; Kreslavski et al., 2009). The nature of the changes in spectral properties induced by LED inter-lighting has not yet been investigated in a high-wire greenhouse tomato canopy.

Canopy inter-lighting is expected to change the photosynthetic rate, but it might also modulate the rate of carbohydrate export from source leaves to sinks. Indeed, recent experiments with LEDs of different wavelengths showed that orange and blue light promoted greater carbohydrate export from the leaves when compared with green or white light (Lanoue et al., 2018); therefore, modulation of the light spectrum by inter-lighting might also affect carbohydrate transport from the lower-canopy leaves in high-wire greenhouse tomatoes. The mechanism driving carbohydrate export from the leaves might include interactions involving phloem and xylem osmotic pressure (Smith and Milburn, 1980; Windt et al., 2006; Nikinmaa et al., 2013), where increased carbohydrate export would be expected when higher rates of transpiration increase the turgor potential gradient between the source and sink (Johnson et al., 1992). However, a recent comparison of the effects of lights of different wavelength on carbohydrate export and transpiration showed no close relationship between these turgor-related traits (Lanoue et al., 2018), indicating an involvement of some other types of regulatory mechanisms. Several enzymes and transporters are considered to act as potential bottlenecks for carbohydrate transport from the leaves (Lemoine et al., 2013), thereby emphasizing the complexity of the regulation of carbon export.

Increases in photosynthesis and carbohydrate export from the leaves in response to LED inter-lighting should increase fruit growth, provided that the sink strength of the fruits is not limiting (Lemoine et al., 2013). One factor that contributes to fruit sink strength is the fruit position in the truss: fruit weights are higher for fruits located proximally in the cluster than for fruits located distally in the cluster. The reason for these differences in weight between proximal and distal fruits under conditions of limited assimilate supply is mainly related to the larger number of cells in the proximal fruits—a situation that arises due to competition for assimilates during floral development (Bertin et al., 2002). Cell division is considered to be the main limiting factor for fruit growth, while cell enlargement at later developmental stages can also be important (Bertin, 2005). Cell division in tomato fruits ends at about 20 days after pollination (Bertin, 2005), so supplementary LED inter-lighting might have only a limited effect on assimilate delivery to flowers and young fruits located at high canopy positions. However, the inter-lighting could significantly contribute to fruit growth during the “enlargement” stage. The fruits at different positions in the clusters could have different sink capacities, defined by their cell numbers, so the effect of inter-lighting might differ for each fruit position in the cluster. A better understanding of the specific effects of LED inter-lighting on individual fruit weights in relation to fruit positions in the clusters might provide insights into the processes that determine fruit weight. This knowledge could lead to management practices that would decrease the variation in size observed among individual fruits, as this is an important marketing consideration.

Increases in photosynthesis in the leaves in the lower and middle parts of the canopy in response to supplemental LED inter-lighting could increase the availability of assimilates for the roots, thereby modulating root activity (e.g., root pressure), since the leaves at the bottom of the canopy are the main source of assimilates for the roots in many plant species (Wardlaw, 1990). Root pressure is viewed as the engine for driving water and nutrients to the shoots during the night, when transpiration is low (Steudle, 2001). The water flux driven by root pressure during the night is important for supplying Ca^2+^ to low-transpiring organs, such as tomato fruits, and is responsible for decreasing blossom end rot (BER) disorders in tomatoes (Taylor and Locascio, 2004).

Roots are also important sources of plant hormones; indeed, many hormones are present in the xylem sap and the importance of xylem transport of hormones is well recognized (Jackson, 1997; Schachtman and Goodger, 2008). This raises the possibility that the aging and decreased photosynthesis observed in the lower canopy leaves not supplemented by LED inter-lighting might not be a direct function of low light intensity and/or an altered FR:R ratio, but they may also represent an indirect effect arising from changes in the hormone composition of the xylem sap. For example, a reduced rate of cytokinin transport to shaded leaves could promote leaf senescence (Boonman et al., 2007).

Both shoot and root activities contribute to fruit growth (Guichard et al., 2001). In particular, water flux from the phloem and xylem makes the biggest contribution to fruit growth because the mature tomato fruits have a water content of 92–95% (Guichard et al., 2001). The extent of the water contribution from the phloem and xylem to the fruits depends on the light conditions: the phloem is the main source during the day, whereas the xylem is the main source during the night (Hossain and Nonami, 2010; Hanssens et al., 2015). Therefore, the effects of supplemental LED inter-lighting on tomato fruit growth may reflect a modulation of both shoot and root activities and subsequent changes in the diurnal rhythm of fruit growth.

The indications that supplemental inter-lighting modulates plant activities ranging from photosynthesis to sink strength of individual fruits in a truss, modulation of root function, and diurnal fruit growth dynamics—and the complexity of the interrelationships between these processes—suggest that an integrative, parallel approach is required to investigate these activities to generate insights into plant responses to LED inter-lighting. Here, we hypothesize that supplemental LED inter-lighting has several effects: i) it changes the light spectrum along canopy and increases plant photosynthesis; ii) it induces an assimilate excess, thereby reducing the competition between fruits in a truss, iii) it modulates root activity (specifically, it changes the hormone composition of xylem sap); and iv) it affects fruit growth dynamics and quality of fruits.

## Materials and Methods

### Plant Materials and Growth Conditions

Two experiments were conducted in an industrial greenhouse in southwestern Norway (58°42’49.2”N 5°31’51.0”E), one from 22 March 2016 to 28 September 2016 (12^th^ to 39^th^ week) and the other from 7 August 2016 to 24 February 2017 (31^st^ week in 2016 to 8^th^ week in 2017). The tomato plants (*Solanum lycopersicum*, Flavance F1) used in the experiment had 12 leaves and the first flowering truss and were planted on 22 March 2016 and on 7 August 2016. The plants were first grown as a subculture beside the previous tomato culture until the first ripe tomato appeared, and then the previous tomato culture was removed. The tomato plants were cultivated as two-shoot branched plants, where the primary shoot had about six trusses more than the second shoot. The final plant density was 1.5 plants (3 shoots) per square meter. The optimal conditions for tomato cultivation in Norway were previously identified in an optimization experiment for year-round cultivation of tomato under artificial light (Verheul et al., 2012).

**Figure 1:**
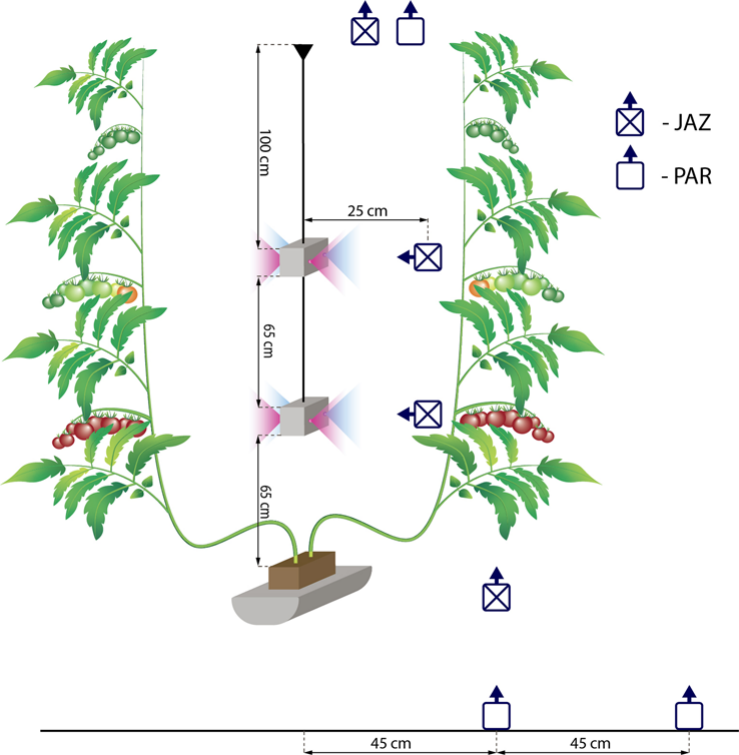
Schematic diagram of the installation of the light-emitting diode lamps and the positions and orientations of the LP9021 PAR sensor used to measure light intensity (PAR) and the JAZ-COMBO (Ocean Optics) (JAZ) used to determine the spectral properties. The distance between rows was 180 cm. The position and orientation of the light intensity and spectral sensors was the same in the control treatment.

**Figure 2:**
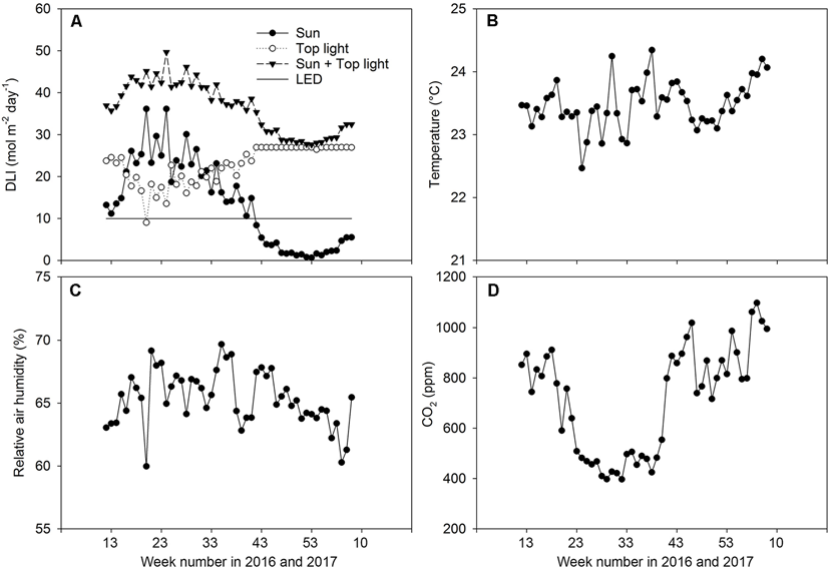
Climate conditions in the greenhouse. Weekly averages for the daily light integral (DLI) for natural irradiance, supplemental high pressure sodium, and light-emitting diode inter-lighting **(A)**, temperature **(B)**, relative air humidity **(C)**, and CO2 concentration in the air **(D)** during experiments.

HPS lamps (SON-T, Gavita, 600 W) with an intensity of 235 W m^−2^ were positioned at about 1.5 m above the top of the canopy and provided 420 µmol m^−2^ s^−1^ at the top of the plant canopy. The top lighting was switch on gradually in the following staggered sequence: first, 1/3 of the lamps were turned on. Fifteen minutes later, another 1/3 of the lamps were turned on. Five minutes later, the remaining lamps were turned on. The LED lamps (Union Power Star, Munich, Germany) with 450 and 660 nm wavelength bands at a diode energy ratio of 20/80 were installed between two branches of the double-shoot plants at two heights (65 and 130 cm from the rockwool block) (**Figure 1**). This provided an additional 70 W m^−2^ of artificial light intensity and the LED lights were switched on for 18 h per day (04:00–22:00). The growth area for the LED-treated plants was 37 m^2^. The HPS lights were switched off automatically when the incoming natural light intensity from outside the greenhouse was more than 250 W m^−2^. The daily light integral (DLI, mol m^−2^ d^−1^) was estimated based on the outdoor global radiation. A conversion factor of 2.2 µmol/J and a light transmission factor of 0.7 for the greenhouse were used. The DLI for artificial light was estimated based on the time that the lamps were switched on and the conversion coefficients were 1.8 and 2.2 µmol/J for HPS and LED lamps, respectively. **Figure 2** shows the DLI from the natural irradiance, the HPS top light lamps, and the LED inter-lighting, as well as the average daily temperature, relative humidity, and CO_2_ concentration during the day for every week of the experiments. The tomato flowers were pollinated by bumblebees. Plants were lowered weekly by about 30 cm, side shoots were removed, and three leaves were removed below the truss with fruits reaching turning stage (Kader, 2011). The trusses were not pruned.

The plants were drip irrigated with a complete nutrient solution based on standardized recommendations containing the following: 17.81 mM NO_3_^−^-N, 0.71 mM NH_4_^+^-N, 1.74 mM P, 9.2 mM K, 4.73 mM Ca, 2.72 mM Mg, 2.74 mM S, and micronutrients with the following concentrations: 15 µmol Fe, 10 µmol Mn, 5 µmol Zn, 30 µmol B, 0.75 µmol Cu, and 0.5 µmol Mo. The electrical conductivity of the nutrient solution was about 2.6 mS cm^−1^ and the pH was 5.8.

### Chlorophyll Index

The chlorophyll index was measured (Hansatech Instruments Chlorophyll Content System CL-01, Norfolk, United Kingdom) on at least 23 uppermost leaves (the first fully developed leaves) and at least 44 low-positioned leaves (close to the second LED lamp from the top). Every leaf was measured three times and the average values were used for statistical analysis.

### Light Properties in the Canopy

Light intensity was measured at the top and at the bottom of the canopy using an LP9021 PAR sensor connected to an HD 9021 logger (Delta OHM SRL, Padova, Italy). At the bottom of the canopy, at least 113 double measurements were done: the first in the middle of the row and the second at 45 cm from the middle of the row (**Figure 1**). The averages of these two measurements were used for statistical analysis.

The spectral properties were analyzed using a JAZ-COMBO device (Ocean Optics, Largo, USA). Measurements were carried out at four levels: at the top of the canopy, at the level of the first LED lamps, at the level of the second LED lamps, and at the bottom of the canopy. In the control treatments, the measurements were carried out at the same height as in the LED inter-lighting treatment (**Figure 1**). During the winter, sunlight did not contribute significantly to the total photosynthetic radiation (**Figure 2**); therefore, measurements were done under conditions without sunlight (evening). These measurements therefore characterized the spectral properties generated by artificial light alone. At least 2 measurements were made at every selected level.

### Daily Fruit Growth

The diurnal changes in fruit diameter were measured over 2–3 days on the second tomato fruits in a truss with a diameter 3–3.4 cm; these fruits were typically located about 40 cm below the tops of the plants. The change in fruit diameter was monitored using the fruit and vegetable dendrometer from Ecomatik and the Dendrometer Data Logger from Ecomatik (DL18, Dachau/Munich, Germany) interfaced to a 4-channel analog Dendrometer Data Logger from HOBO (Onset Computer Corporation, Bourne, USA). The experiment was replicated twice so that 6 fruits were investigated for each treatment. The presented values show the relative increase in fruit diameter every 30 min after min–max normalization (Han et al., 2012) with the assumption that, over a 24 h period, the fruit diameter would increase by 1 U.

### Photosynthesis

The leaf CO_2_ exchange rate was monitored for 2 days simultaneously for two leaves and two treatments using a PTM-48A Photosynthesis Monitor (Phyto-sensor Group, Chisinau, Moldova) with self-clamping leaf chambers (Balaur et al., 2009). The photosynthesis of the low-positioned leaves, located between the top and bottom lamps, was determined from four independent measurements, so the mean photosynthesis values were based on independent measurements of eight individual leaves for every treatment. All measurements were carried out on randomly selected leaves between 20 January 2017 and 19 February 2017. Measurements on single leaves were carried out for 2–3 days. The leaves were exposed to the natural sunlight conditions. Before data analysis, we excluded measurements that had been obtained during periods of strong variations in the air CO_2_ concentration, because this variation directly affects the photosynthesis rate. Upon completion of the photosynthesis measurements, the leaves were removed from the plants and the leaf area was measured using Easy Leaf Area app, Davis, USA (Easlon and Bloom, 2014). These excised leaves were subsequently dried for 48 h at 70°C in an oven and used for dry weight determinations. The specific leaf area (SLA) was calculated by dividing the leaf area by the leaf dry weight.

### Harvest

The growth and fruit yield for both experiments were measured for five plants (10 shoots) in every treatment. Fruits were harvested two times per week. Between January and February 2017, the fruits were harvested and weighed individually and their positions in the truss were recorded. Each position for every treatment was represented by 47 to 70 fruits. Final destructive harvests were performed on 24 February 2017 on four randomly selected double-shoot plants. Dry weights (dried at 70°C for 96 h) of leaves, stems, and fruits were measured.

### Xylem Sap Collection

The rockwool slab was saturated with nutrient solution for xylem sap sampling by the root pressure method (Alexou and Peuke, 2013). The plant was decapitated, leaving a stem about 10 cm long. The cut surface was cleaned with deionized water and a silicon tube was fixed over the stump and sealed with silicone grease. The xylem exudate was collected with a pipette after a 1 h run for a period of 60 min, stored in plastic vials on ice, and subsequently frozen in liquid nitrogen.

### Ion Analysis

Ion composition analysis of fruits and xylem sap was performed at room temperature on an 883 Basic IC plus ion chromatograph (Metrohm, Switzerland) equipped with conductivity detector and 863 compact autosampler (Metrohm, Switzerland). Cations (NH_4_^+^, Na^+^, K^+^, Ca^2+^, Mg^2+^) were separated on a Metrosep C4 column (150 × 4 mm i.d.) protected by a Metrosep C4 guard column (5 × 4 mm i.d.) with an eluent containing 1.7 mM nitric acid and 0.7 mM dipicolinic acid supplied at 1 ml min^−1^. Anions (Cl^−^, NO_3_^−^, PO_4_^3+^, SO_4_^2+^) were determined by suppressed IC using a Metrosep A Supp 5 column (150 × 4 mm i.d.) equipped with a Metrosep A supp 5 guard column (5 × 4 mm i.d.) with eluent comprised of 3.2 mM Na_2_CO_3_, 1.0 mM NaHCO_3_, and 5% (v/v) acetone supplied at 1 ml min^−1^. Background conductivity was suppressed with an MSM A ion suppressor (Metrohm, Switzerland); 0.1 M H_2_SO_4_ and deionized water were used for suppressor regeneration and rinsing, respectively. Eluents were prepared using deionized water obtained from ELGA PURELAB Option-R7 (ELGA Labwater, UK) water purification system, filtered through 0.45 µm nylon filter membranes (VWR, USA), and degassed under vacuum. Prior to analysis, xylem sap was diluted 10-fold and 100-fold with deionized water for cations and anions, respectively, and filtered through 0.2 µm nylon syringe filters (Pall Corporation, USA). Three tomato fruits were homogenized with an immersion blender. The resulting tomato homogenate was centrifuged for 10 min at 2,880×g at room temperature in a Jouan B4 centrifuge (Thermo Fisher Scientific, USA). After centrifugation, the supernatant was diluted 50-fold and 100-fold with deionized water for cations and anions, respectively. Identification and quantification of cations and anions were performed using external calibration (r^2^ > 0.99, RSD < 3%) with multi element analytical standard mixtures for IC (SpectraScan SS-2877S and Sigma-Aldrich 69734, respectively) using MagIC Net ver. 3.2 software (Metrohm, Switzerland).

### Quality Analysis of Fruits

Soluble solid content (expressed as ^°^Brix) was measured with a PR-101α digital refractometer (ATAGO, Japan). Total titratable acidity was determined using a 794 Basic Titrino automatic titrator (Metrohm, Switzerland) and expressed as citric acid equivalents (grams of CAE per 100 g fresh weight).

### Hormone Analysis

A 500 µl volume of xylem sap were filtered through a 10,000 molecular weight cut-off centrifugal filter unit (Amicon Ultra 0.5, Merck, USA) according to the supplied user guide. A 200 µl sample of filtrate was then freeze-dried (BK-FD10S; BIOBASE, China). Samples were purified and analyzed using a previously described method (Dobrev and Kaminek, 2002; Dobrev and Vankova, 2012). The samples were dissolved in cold (−20°C) methanol/water/formic acid (15/4/1 v/v/v) and the following stable isotope-labeled internal standards (10 pmol/sample) were added: ^13^C_6_-IAA (Cambridge Isotope Laboratories, USA); ^2^H_4_-SA (Sigma-Aldrich); ^2^H_3_-PA, ^2^H_3_-DPA (NRC-PBI); ^2^H_6_-ABA, ^2^H_4_-7OH-ABA, ^2^H_5_-ABA-GE, ^2^H_2_-OxIAA, ^2^H_5_-JA, ^2^H_2_-GA_4_, ^2^H_2_-GA_8_, ^2^H_2_-GA_19_, ^2^H_2_-GA_20_, ^2^H_5_-transZ, ^2^H_5_-transZR, ^2^H_5_-transZ7G, ^2^H_5_-transZ9G, ^2^H_5_-transZOG, ^2^H_5_-transZROG, ^2^H_5_-transZRMP, ^2^H_3_-DZ, ^2^H_3_-DZR, ^2^H_3_-DZ9G, ^2^H_7_-DZOG, ^2^H_3_-DZRMP, ^2^H_6_-iP, ^2^H_6_-iPR, ^2^H_6_-iP7G, ^2^H_6_-iP9G, and ^2^H_6_-iPRMP (Olchemim, Czech Republic). Two hormone fractions were extracted with a reverse phase–cation exchange solid-phase extraction column (Oasis-MCX, Waters, UK): the acid fraction was eluted with methanol (auxins, ABA, SA, JA) and the second fraction was eluted with 0.35 M NH_4_OH in 60% methanol (CKs, ET). Fractions were analyzed by HPLC (Ultimate 3000, Dionex, USA) coupled to the 3200 Q TRAP hybrid triple quadrupole/linear ion trap mass spectrometer (Applied Biosystems, USA). The hormones were quantified by the isotope dilution method with multilevel calibration curves (r^2^ > 0.99). Data processing was carried out with Analyst 1.5 software (Applied Biosystems, USA).

### Statistics

One-factor repeated-measures analysis of variance (ANOVA) was used to identify the significant differences in yield, number of trusses, individual fruit weights, and number of fruits per truss based on two experiments. Data for light interception and FR:R ratio, SLA, destructive harvest of biomass, and ion and phytohormone compositions were statistically analyzed by one-way ANOVA. Data for chlorophyll index, PLUE, and fruit relative growth rate were analyzed by two-way ANOVA. If the two-way ANOVA showed significant main effects and interaction, the Student’s t-test was used to determine the significant differences among the means. Variations in individual fruit weights were estimated using the coefficient of variation (CV) expressed as a percentage of the standard deviation of the mean (Clewer and Scarisbrick, 2001). Data were statistically analyzed using the Statistica 13 software package (TIBCO, USA).

## Results

### Biomass and Yield Components

Based on the analysis of two experiments, the supplemental LED inter-lighting increased the tomato fruit yield by 21% (**Table 1**). The main yield components contributing to this greater yield were increases in the mean weight of the tomato fruits (8.5%) and accelerated plant development, as indicated by the larger number of trusses per plant (9.9%); however, the fruit number per truss changed only slightly (**Table 1**). The supplemental LED lighting also increased the plant aboveground biomass (17%) due to a greater weight of leaves (26%) and stems (20%) at the final harvest (**Table 2**).

**Table 1:** The effects of supplemental light-emitting diode inter-lighting on mean values for yield, fruit weight, number of trusses, and number of fruits per truss from two greenhouse experiments.

Traits	Treatments		Percentage increase %
	Control	LED	
Yield, kg m^−2^	30.8a	37.2b	20.8
Fruit weight, g	106.5a	115.5b	8.5
Number of trusses	34.3a	37.7b	9.9
Number of fruits per truss	5.6a	5.7b	1.8

Significance was confirmed by one-factor repeated-measures analysis of variance (ANOVA) at p < 0.01. Means with different letters are significant different.

**Table 2:** The effect of supplemental light-emitting diode inter-lighting on biomass, fruit weight, number of trusses, and leaf and stem weights of greenhouse tomato plants after 18 weeks of harvesting (mean ± SD) in experiment 2.

Traits	Treatments		p
	Control	LED	
Biomass at the final harvest, g plant^−1^ (n = 4)	820 ± 70	956 ± 16	0.009*
Mean tomato fruit weight, FW g (n > 400)	110 ± 25	116 ± 21	0.000*
Number of trusses per plant (n = 4)	32.8 ± 1.5	36.5 ± 2.4	0.037*
Leaves, DW g (n = 4)	146 ± 13	184 ± 22	0.025*
Stem, DW g (n = 4)	335 ± 34	402 ± 44	0.052

*indicate signiﬁcant differences at p < 0.05.

### Light Properties in the Tomato Canopy

**Figure 3:**
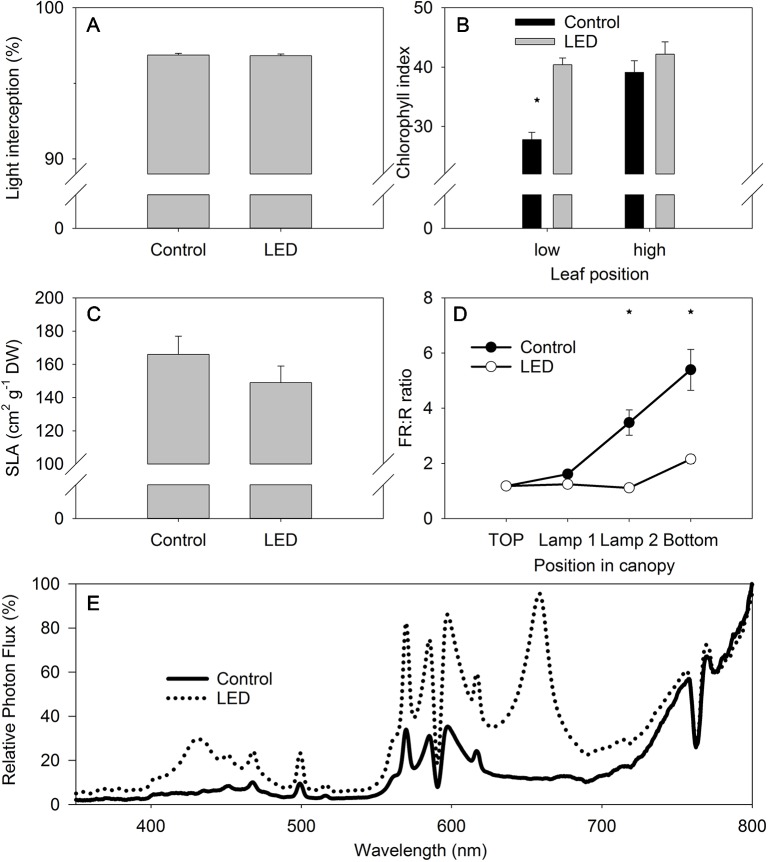
The effect of supplemental light-emitting diode (LED) inter-lighting on light interception in the canopy (n = 113–115, bars represent SE **(A)**. Chlorophyll index (CL-01 units) of the high- and low-positioned leaves, bars represent SE **(B)**. Specific leaf area (SLA) (cm^2^ g^−1^), bars represent SE **(C)**. Ratio of far-red to red light, bars represent SE **(D)**. Modulation of the light spectrum between 350 and 800 at the bottom of the canopy **(E)**. The effect of LED and leaf position on chlorophyll index was analyzed using two-factor analysis of variance (ANOVA). The first factor was the LED effect (LED) and the second factor was leaf position (P). Analysis of the source of variation (ANOVA) showed that the effect of LED was significant at p < 0.001; leaf position was significant at p < 0.001; and the interaction between LED × P was significant at p < 0.01. Stars indicate significant differences at p < 0.05.

No differences in the light intensity were observed at the bottom of tomato canopy, despite the LED inter-lighting (**Figure 3A**). The absence of a difference in intensity was attributed to the higher light interception by the low-positioned leaves receiving LED light. These LED-light receiving leaves in the lower part of the canopy were greener than similarly positioned, non-LED-supplemented control leaves, as confirmed by non-destructive chlorophyll measurements (**Figure 3B**). The leaves of the plants receiving LED light also tended to be thicker, as indicated by the lower SLA values (**Figure 3C**), which again could contribute to the higher chlorophyll values. Nevertheless, the difference in SLA between the LED-treated and control plants was not statistically significant.

The light intensity at the bottom of the canopy did not differ significantly for plants with and without LED inter-lighting (**Figure 3A**); however, the FR:R ratio was more than two-fold lower underneath plants with LED inter-lighting than underneath plants without LED inter-lighting (**Figure 3D**). This indicates that the FR:R ratio might be an important signal that triggers differences in leaf activities between canopies with and without LED inter-lighting. Spectral analysis at the bottom of the canopy showed that the LED inter-lighting not only changed the FR:R ratio, but it also introduced a blue light peak (400 to 500 nm) that was absent from the control conditions (**Figure 3E**).

### Diurnal Photosynthetic Light Use Efficiency

**Figure 4:**
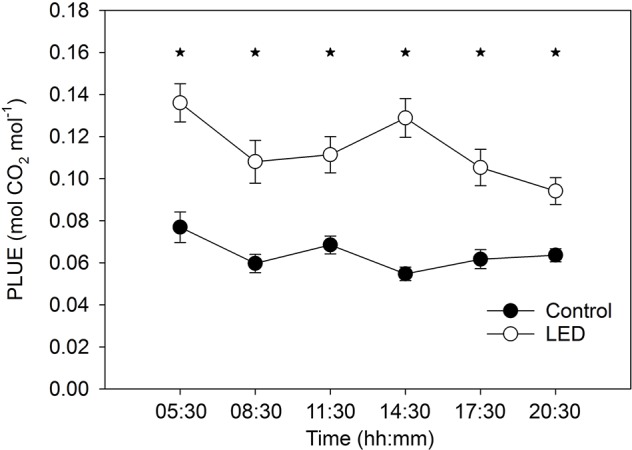
The effect of the supplemental light-emitting diode (LED) inter-lighting on photosynthetic light use efficiency (PLUE). Every mean shown is based on 41–72 measurements. Bars represent SE. Stars indicate significant differences at p < 0.05. The experiment was analyzed using two-factor analysis of variance (ANOVA). The first factor is the LED effect (LED) and the second factor is time (T). Analysis of the source of variation (ANOVA) showed that the effect of LED was significant at p < 0.001; time was significant at p < 0.05; and the interaction between LED × T was significant at p < 0.05.

The photosynthetic light use efficiency (PLUE) was, on average, two-fold higher for the leaves receiving LED light than for the control leaves (**Figure 4**). In addition, the LED-treated plants revealed an interaction between light treatment and time, as indicated by a reduction in PLUE to 65% at the end of the day. By contrast, the control plant leaves maintained a constant PLUE throughout the day (**Figure 4**).

### Root Activity

Plants with LED inter-lighting exuded more xylem sap when compared to control plants (**Table 3**). Analysis of the nutrients in the xylem sap revealed a lower concentration of most ions in the plants supplemented with LED inter-lighting than in the control plants (**Table 3**).

**Table 3:** The effect of supplemental light-emitting diode inter-lighting on exudation and composition of xylem sap in greenhouse tomato plants at final harvest (mean ± SD; n = 3 for Control, n = 4 for LED).

Traits	Treatments		p
	Control	LED	
Xylem sap exudation, ml h^−1^	14.1 ± 2.2	20.3 ± 2.5	0.020*
**Anions** (mM)	60.6 ± 4.1	41.8 ± 3.8	0.001*
Chloride (Cl^−^)	2.96 ± 0.63	2.92 ± 0.27	0.928
Nitrate (NO_3_^−^)	42.5 ± 4.6	26.5 ± 4.8	0.006*
Phosphate (PO_4_^3+^)	5.09 ± 0.12	3.97 ± 0.79	0.063
Sulfate (SO_4_^2+^)	4.95 ± 0.26	4.12 ± 0.39	0.026*
**Cations** (mM)	51.3 ± 5.6	39.9 ± 1.9	0.011*
Ammonium (NH_4_^+^)	1.00 ± 0.18	0.63 ± 0.06	0.011*
Potassium (K^+^)	21.6 ± 2.1	16.5 ± 2.1	0.022*
Sodium (Na^+^)	0.48 ± 0.10	0.28 ± 0.05	0.017*
Calcium (Ca^2+^)	10.62 ± 1.21	8.79 ± 0.35	0.031*
Magnesium (Mg^2+^)	3.49 ± 0.66	2.48 ± 0.24	0.033*
Total ions (mM)	111.9 ± 9.3	81.7 ± 5.3	0.003*
**Phytohormones** (nmol)			
ABA	7.2 ± 3.7	9.3 ± 2.9	0.823
SA	40 ± 6	34 ± 6	0.237
JA	68 ± 29	251 ± 59	0.005*
JA-Ileu	4 ± 2	24 ± 23	0.211
cisOPDA	2.1 ± 0.7	3.3 ± 2.7	0.505
tZ	0.22 ± 0.14	0.29 ± 0.09	0.443
IAA	0.33 ± 0.22	0.34 ± 0.29	0.956

ABA, abscisic acid; SA, salicylic acid; JA, jasmonic acid; JA-Ileu, JA-isoleucine, cisOPDA (JA precursor); tZ, trans-zeatin; IAA, indole-3-acetic acid. *indicate significant differences at p < 0.05.

Analysis of xylem sap for all major plant hormones showed that LED inter-lighting increased jasmonic acid (JA) levels more than three-fold above the control levels (**Table 3**). LED inter-lighting had no significant effects on the concentrations of other hormones.

### Diurnal Dynamic of Fruit Growth

**Figure 5:**
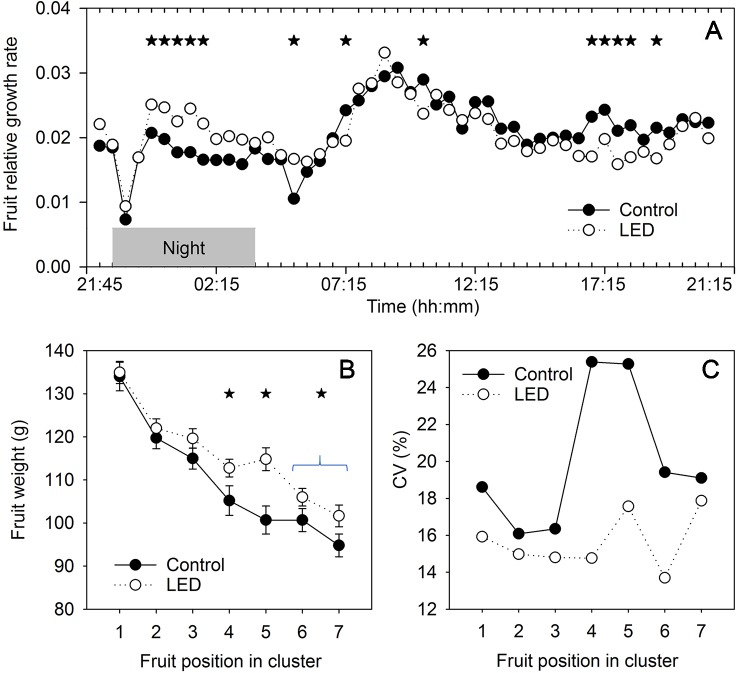
The effect of supplemental light-emitting diode (LED) inter-lighting on fruit growth. **(A)** Relative changes in fruit diameter during the day. Every mean is based on 24 and 17 measurements for the control and LED treatments, respectively. The experiment was analyzed using two-factor analysis of variance (ANOVA). The first factor is LED effect (LED) and the second factor is time (T). Analysis of the source of variation (ANOVA) showed that the effect of LED was not significant, T was significant at p < 0.001, and interaction between LED × T was significant at p < 0.001. Stars indicate significant differences at p < 0.05. The effect of LED and fruit position in the truss on mean fresh fruit weight **(B)** and the coefficient of variation (CV) for fresh fruit weight **(C)**. Bars represent standard error (SE). Stars indicate significant differences at p < 0.05. Clusters with fewer than 6 tomatoes were excluded from analysis. The mean and CV for every cluster and every treatment were based on 43–60 measurements.

In the control treatment, the relative growth rate of the fruits was lower during the night than during the day. We found two short periods of declines in fruit growth, one at 0.5 h at the beginning of the night and the other at 1.5 h at the beginning of the day (**Figure 5A**). The highest relative fruit growth rate was observed 5 h after switching the lights on. Two-way ANOVA showed a significant interaction between light and time factors, which indicated a difference in the diurnal patterns of fruit growth between the plants receiving LED light and the control plants. Supplemental LED inter-lighting enhanced the relative fruit growth rate during the night and reduced it at the end of the light period (**Figure 5A**).

### Fruit Size and Fruit Quality

In the control treatment, the fruit size decreased by 29% from the proximal to the distal position in the truss (**Figure 5B**). LED inter-lighting did not change the weight of the fruits in the basal positions of the truss; however, it increased the sizes of the fruits in the middle positions and tended to increase the size of distal fruits. Interestingly, the mean size of the fruits was also normalized by LED inter-lighting, as the individual fruits in a truss showed less variation in size (**Figure 5C**), resulting in a more even fruit size across the truss. This effect was strongest in the middle-positioned fruits in the trusses.

A key quality parameter, the soluble solid content (SSC) value, was not changed by LED inter-lighting (**Table 4**). Total titratable acidity (TTA) increased by 9% in response to LED inter-lighting, but this difference was not statistically significant. Ion chromatography (IC) analysis of the fruits showed higher ammonium (31%) and lower sodium (30%) ion concentrations in the fruits from plants receiving LED inter-lighting than in the control fruits. Supplemental lighting also increased the Ca concentration (62%) in the fruits; however, this effect was not statistically significant.

**Table 4:** The effect of supplemental light-emitting diode inter-lighting on ion concentrations, soluble solid content (SSC), and total titratable acidity (TTA) of fresh tomato fruits (mean ± SD, n = 7 for the control, and n = 6 for LED-supplemented plants).

Traits	Treatments		p
	Control	LED	
**Anions** (mM)			
Chloride (Cl^−^)	5.63 ± 0.91	5.68 ± 0.53	0.909
Nitrate (NO_3_^−^)	0.167 ± 0.22	0.247 ± 0.10	0.438
Phosphate (PO_4_^3+^)	6.10 ± 0.83	6.19 ± 0.47	0.814
Sulfate (SO_4_^2+^)	1.36 ± 0.16	1.50 ± 0.17	0.136
**Cations** (mM)			
Ammonium (NH_4_^+^)	0.85 ± 0.11	1.11 ± 0.22	0.019*
Potassium (K^+^)	60.0 ± 4.5	63.7 ± 6.1	0.236
Sodium (Na^+^)	1.28 ± 0.16	0.89 ± 0.21	0.003*
Calcium (Ca^2+^)	0.13 ± 0.13	0.21 ± 0.05	0.192
Magnesium (Mg^2+^)	2.67 ± 0.19	2.82 ± 0.35	0.380
SSC (°Brix)	4.36 ± 0.34	4.38 ± 0.18	0.870
TAA (g citric acid/100 g tomato)	0.556 ± 0.044	0.607 ± 0.045	0.070

*indicate significant differences at p < 0.05.

## Discussion

Supplementation of greenhouse tomato plants with LED inter-lighting increased plant productivity (**Table 1**), biomass (**Table 2**), and photosynthesis in the lower leaves (**Figure 4**). However, light does not serve only as the energy source for photosynthesis; it is also a signal that controls morphological and physiological changes in plant growth and development to allow modulation of plant responses to changes in ambient light conditions. In agreement with this, our results show a complex effect of inter-lighting on both shoot and root activities and their interactions. The end result was increased fruit yields in greenhouse tomato plants.

### Supplemental Light-Emitting Diode Inter-Lighting Modulates the Light Spectrum and Increases Photosynthetic Light Use Efficiency and Light Absorption by the Canopy

Supplemental LED lighting strongly modulated the light spectral composition from the top to the bottom of the tomato canopy by reducing the FR:R ratio when compared to ratio in the control unsupplemented treatment (**Figure 3D**). The new light properties enhanced plant source capacity due to the higher PLUE and due to better light absorption in the tomato canopy. The higher PLUE is evident from the long-term gas-exchange measurements obtained with the gas-exchange monitoring system (PTM-48A) (**Figure 4**). The higher light absorption is evident from the observation that, even with the supplemental LED inter-lighting, the same light intensity was found at the bottom of the canopy in both the inter-lighting and control conditions (**Figure 3A**).

Our results showing that LED inter-lighting increases PLUE agree with the findings of other investigations that have shown positive effects of LED lighting on photosynthesis of lower-canopy leaves (Pettersen et al., 2010; Gomez and Mitchell, 2016). These previous studies reported photosynthesis increases by as much as two-fold, in agreement with our observations (**Figure 4**). The LED lighting also increased the greenness of the low-positioned leaves, indicating a possible delay in their senescence—senescence was clearly evident in the control plants in leaves at similar lower-canopy positions (**Figure 3B**). This delay in senescence in the LED-treated lower leaves might be related to the exposure of these leaves to a higher light intensity and/or to a modified light spectrum during their development (Liebsch and Keech, 2016).

The strong alteration of the light spectrum by LED inter-lighting, namely the lower FR:R ratio and the presence of a blue peak (**Figure 3E**), indicates that the modified light spectrum might contribute to the observed alterations in leaf properties, such as increased leaf greenness and PLUE. Interestingly, despite the large difference in the light spectrum at the bottom of the tomato canopy, the light intensity itself was the same beneath the plants regardless of light treatment (**Figure 3A**). This absence of a difference was due to the higher light absorption along the canopy, as inferred by the more intense greenness of the leaves of the plants receiving LED light (**Figure 3B**). Note, however, that measuring light intensity beneath the canopy (**Figure 3A**) does not estimate the real light intensity to which low-positioned leaves were exposed. We also cannot exclude the possibility that the increased light intensity due to supplemental LED inter-lighting directly affected leaf properties, such as leaf greenness and PLUE. The complexity is also increased by the fact that leaf orientation can be modulated by light to increase light interception (Sarlikioti et al., 2011) and this will ultimately affect the light distribution in the canopy and the light interception by the canopy (de Visser et al., 2014). Moreover, LED lamps with different spectra can also modulate the leaf angle to affect light absorption at the leaf and crop levels (Dieleman et al., 2019).

### Light-Emitting Diode Inter-Lighting Induces Assimilate Excess and Differentially Regulates Fruit Growth At Different Positions in the Truss

Photosynthesis decreases at the end of a day if leaves accumulate an excess of photoassimilates that cannot be exported from the leaves (Matthews et al., 2017); this is especially an issue if the plants are sink limited. In our study, the LED inter-lighting caused a reduction in PLUE at the end of the day (**Figure 4**), indicating that low-positioned leaves accumulated an excess of assimilates. Despite this excess production of assimilates, the LED inter-lighting did not stimulate the growth of basal fruits in the truss but instead increased the growth of middle-positioned and distal fruits (**Figure 5B**).

The differential effect of inter-lighting on fruits at different truss positions should be considered in light of the presence of two major stages of fruit development. The first stage includes the first 10–14 days after pollination, when the cells in the fruits are actively dividing and the potential sink capacity is defined, whereas the second stage is associated with fruit cell enlargement (Bungerkibler and Bangerth, 1982). The first stage usually has the greatest impact on the differences in tomato sizes between proximal and distal fruits (Bertin et al., 2002), and this can explain why, under the LED inter-lighting treatment (i.e., a condition that was not source limited), the proximal fruits were still larger than the distal fruits (**Figure 5B**). We did not expect that inter-lighting would enhance the sink capacity of the fruits, because young tomato fruits are located at the top of the canopy, and the light from inter-lighting might not reach the top leaves that supply most of the assimilates to these fruits. By contrast, fruits undergoing cell enlargement are located in the middle and bottom parts of the canopy, so the light conditions for the middle and low leaves might play a major role in determining the ultimate supply of carbohydrates for the enlarging fruits during the second stage of fruit development. Therefore, inter-lighting was expected to contribute mainly to fruit enlargement but would not increase the sink capacity of the fruits.

In this context, the absence of a positive effect of inter-lighting on the weight of fruits in the proximal position in the truss indicates that the size of these fruits was limited by their sink capacity (**Figure 5B**). Consequently, any additional assimilates produced in response to supplemental LED did not change the sizes of these fruits. By contrast, the significant increase in fruit weight observed in the middle and distal positions of the truss indicates that the sink capacity of these fruits was not completely utilized in the control treatment. Therefore, an increased availability of assimilates during the filling stage increased the final size of the fruits grown with supplemental LED inter-lighting. Importantly, this additional contribution of photoassimilates also decreased the variation in size among the fruits (**Figure 5C**), which is an important parameter for tomato fruit market value.

### Supplemental Light-Emitting Diode Inter-Lighting Modulates Root Activity

The improved PLUE of low-positioned leaves might be the source of the supplemental supply of carbohydrates that is used for fruit growth during their filling stage; however, this increased photoassimilate supply may also affect root activities. Indeed, the lower leaves that were most affected by the supplemental LED inter-lighting are also the leaves that are the most responsible for supplying assimilates to the roots (Wardlaw, 1990). Therefore, we investigated whether supplemental LED inter-lighting might be altering root activity. Our results confirmed that LED inter-lighting modulates root activity, as indicated by the increased rate of xylem sap exudation from decapitated plants, by the lower concentration of ions in exuded xylem sap, and by the modulation of the hormonal composition of the xylem sap (**Table 3**). Analyses of the fluxes and ion concentrations of xylem sap from decapitated plants give estimations of the cell-to-cell pathway of water transfer from the nutrient solution to the xylem lumen (Lopez et al., 2003). In this cell-to-cell pathway, the water volume flux is predominantly driven by root pressure, i.e., the osmotic gradient between the nutrient solution and xylem lumen.

The observed higher rate of xylem sap flux under LED treatment (**Table 3**) can be explained by the higher amount of carbohydrates produced by the supplemental LED inter-lighting and transported to the roots. A close relationship has been demonstrated between root pressure and the carbohydrate status of the roots (Marschner, 1995). However, analysis of the diurnal pattern of xylem sap flux in young maize plants showed circadian regulation, with two peaks (one in the daytime and one at night), indicating that the regulation might be more complex, where light acts as a signal (Lopez et al., 2003).

The higher rate of xylem sap flux at lower ion concentrations in xylem (i.e., the smaller osmotic pressure gradient between xylem and external nutrient solution) shown in **Table 3** indicates that the LED-treated plants had a higher root permeability to water. Previous investigations with several species, including tomato, have shown that root permeability is enhanced by JA (Sanchez-Romera et al., 2014). In the context of this knowledge, our results, indicating a strongly increased JA concentration in the xylem sap of LED-treated plants, suggest a possible regulatory role for JA in the increased root permeability observed in the LED treatment. Other plant hormones, such as ABA, also increase root permeability (Kudoyarova et al., 2011); however, in our experiment, LED did not affect the ABA concentration in the xylem sap (**Table 3**). Therefore, we assumed that ABA was not involved in the improved root conductance observed in response to supplemental LED inter-lighting.

One unanswered question is how LED inter-lighting might regulate JA levels in xylem sap. Cargnel and co-workers (Cargnel et al., 2014) demonstrated the possibility of cross-talk between phytochrome B and JA signaling; therefore, we assume that the modification in the spectral properties at the bottom of the canopy and consequent phytochrome activation were likely responsible for the increased JA levels in the xylem sap and the subsequent increase in root pressure. However, confirming this and identifying the underlying mechanism will require further investigation.

The main source of the regulation of root permeability are the aquaporins, which direct the passive movement of water from the ambient solution into the xylem (Lopez et al., 2003; Postaire et al., 2010), as indicated by studies showing a correlation between water flux and the levels of aquaporin proteins (Lopez et al., 2003). Moreover, *Arabidopsis* knockout mutants of the aquaporin PIP2-2 exhibited a decrease in sap flux when compared to wild type and an increase in sap osmolarity (Javot et al., 2003). JA is assumed to regulate the activities of aquaporins by phosphorylation (Sanchez-Romera et al., 2014).

### Fruit Growth and Fruit Composition Is Affected by Inter-Lighting by Modulation of Root Activity

Our observations that LED inter-lighting increases the root pressure and that LED-treated plants have higher relative fruit growth rates during the night suggest that fruit growth is affected by modifications of root activity that occur in response to LED lighting. Indeed, root pressure is viewed as the main power driving the water supply that contributes to fruit growth during the night (Hossain and Nonami, 2010; Hanssens et al., 2015). Therefore, we assume that a higher root pressure contributes to the ultimately higher fruit weight of fruits in the LED-treated plants. Root pressure during the night is important for the transport of nutrients as well as water. However, the nutrient concentration in the xylem sap was lower in LED-treated plants than in the control for all nutrients, with the exception of chloride (**Table 3**). Therefore, the total flux of nutrients to the aboveground parts should be the same in both treatments and should result in the same concentrations of most ions in the fruits (**Table 4**). Only sodium showed a lower concentration in the light-supplemented plants; however, lower sodium levels are unlikely to have negative effects on plant growth since sodium is not an essential nutrient. By contrast, the fruits of LED-treated plants had higher ammonium levels and tended to have higher Ca^2+^ concentrations. Calcium is a nutrient with low phloem mobility and is transported into the fruits primarily in the xylem (Marschner, 1995). A positive effect of Ca^2+^ against BER disorders was not apparent in our experiment because neither the control nor the light-supplemented plants displayed any signs of BER. However, the observation of increased xylem flux during the night and the trend toward an increased Ca^2+^ concentration in the fruits indicate a potential positive effect of LED inter-lighting against the development of BER disorders.

Interestingly, although LED inter-lighting increases fruit growth during the night (**Figure 5A**), when root pressure is the main contributor to growth, the fruits showed the same SSC (Brix) under both light conditions, indicating no dilution effect due to a higher xylem input during the night. This assumes that more sucrose, the main transport sugar, was transported during the day, which might reflect a higher sucrose concentration in the phloem. Indeed, increased light intensity can increase the concentration of sucrose in phloem sap (Xu et al., 2018). Therefore, despite the relatively lower fruit growth during the day under the LED treatment, a higher phloem sucrose concentration might compensate for any carbohydrate dilution due to the higher water flux during the night.

A greater transport of sucrose might be regulated by transpiration in the LED-treated leaves, as both the hydrostatic tension of water in leaf xylem and the osmotic pressure in leaf phloem defines the turgor pressure that drives phloem transport (Smith and Milburn, 1980; Johnson et al., 1992; Windt et al., 2006). However, a comparison of the effects of light of different wavelengths revealed no close relationship between sucrose transport and transpiration, indicating that sucrose export cannot be explained solely by modulation of leaf turgor pressure (Lanoue et al., 2018). The most promising key player candidates for the regulation of sucrose transport are sucrose transporters (SUT) (Griffiths et al., 2016). The post-transcriptional upregulation of SUT1 by light in tomato plants (Xu et al., 2018) assumes a contribution in the regulation of sucrose transport under conditions of supplemental LED inter-lighting.

In conclusion, supplemental LED inter-lighting does more than just increase the light intensity experienced by the greenhouse tomato crop. It also alters the spectral properties of the impinging light, thereby activating phytochrome signaling in the leaves at low canopy positions. This suppresses leaf senescence and increases the PLUE of these lower leaves, thereby increasing overall plant growth and improving the assimilate supply during fruit filling. In addition, supplemental LED inter-lighting enhances root pressure, which increases water supply to the fruits and subsequently promotes fruit growth during the night.

## Data Availability Statement

The raw data supporting the conclusions of this article will be made available by the authors, without undue reservation, to any qualified researcher.

## Author Contributions

IP and MV design the experiment. IP and MP wrote the paper. IP and MP performed the experiments and most analysis. DK analyzed ion concentrations. JL analyzed hormone concentration.

## Conflict of Interest

The authors declare that the research was conducted in the absence of any commercial or financial relationships that could be construed as a potential conflict of interest.
